# Effects of different types of xanthophyll extracted from marigold on pigmentation of yellow-feathered chickens

**DOI:** 10.5713/ab.23.0097

**Published:** 2023-06-26

**Authors:** Yu Wei, Kun Qin, Xu Qin, Fulong Song, Xiao Xu

**Affiliations:** 1 Hubei Key Laboratory of Animal Nutrition and Feed Science (Wuhan Polytechnic University), School of Animal Science and Nutritional Engineering, Wuhan Polytechnic University, Wuhan 430023, China

**Keywords:** Marigold, Monohydroxyl Pigment, Pigmentation, Xanthophyll, Yellow-Feathered Chicken

## Abstract

**Objective:**

This study aimed to explore the effects of different types of xanthophyll extracted from marigold on the growth performance, skin color, and carcass pigmentation.

**Methods:**

A total of 192 healthy 60-day-old yellow-feathered broilers weighing an average of 1,279±81 g were randomly allocated to 4 groups, each with 6 replicates and 8 broilers. The 4 treatments were as follows: i) CON group, fed with basal diet; ii) LTN group, supplemented with lutein; iii) MDP group, supplemented with monohydroxyl pigment including dehydrated lutein, β-cryptoxanthin, and α-cryptoxanthin; iv) LTN+MDP group, supplemented with lutein and monohydroxyl pigment in proportion to 1:1. The supplementary content of LTN, MDP, and LTN+MDP was 2 g/kg. Skin color was measured after 7, 14, 21, and 28 days of feeding the dietary treatments. The breast, thigh, and abdominal fat of slaughtered chickens were stored in cold storage at 4°C for 24 hours and then the meat color of lightness (L*), redness (a*), and yellowness (b*) values was determined.

**Results:**

The results showed that all treatments enhanced the yellow scores of subwing skin on day 14, 21, and 28 (p<0.05), and the mixture of lutein and monohydroxyl pigment promoted the yellow scores of shanks on day 14, 21, and 28 (p<0.05). The mixture of lutein and monohydroxyl pigment increased the yellow scores of beaks and all treatments enhanced the yellow of shanks on day 28 (p<0.05). In addition, all treatments improved the yellow (b*) values of breast and thigh muscle, moreover, the monohydroxyl pigment and the mixture of lutein and monohydroxyl pigment enhanced the values of redness (a*) and yellow (b*) of abdominal fat (p<0.05).

**Conclusion:**

In summary, different types of xanthophyll extracted from marigold significantly increased the yellow scores of skin color and the yellow (b*) values of carcass pigmentation. Especially, the mixture of lutein and monohydroxyl pigment was more efficient on skin color.

## INTRODUCTION

The color of skin and pigmentation of muscle are decisive factors in determining the preference of consumers and evaluating the meat quality of broilers in many cosmopolitan regions [1,2]. However, it cannot be synthesized in animal bodies, so it must be taken from feed [3], but the quantity and types of pigments provided by corn and soybean-based commercial diets don’t satisfy the preference of consumers for yellow-feathered broiler products [4]. Therefore, to meet the consumer’s favour and market demand, the pigment has been applied as a feed additive for improving the color of broiler and aquaculture animal products which is the result of the pigment deposited into tissues [5]. The pigment usually includes a natural and a synthetic pigment, which is more steady and lower in price compared with natural pigment, nonetheless, the safety and bio-availability of synthetic pigment has been challenged [6]. A large number of works have indicated that carotenoid-enriched products have potential to improve the safety and bio-availability than synthetic pigment [7,8].

During the past several decades the premier natural pigment additive sources of carotenoids in poultry feed included yellow corn, dehydrated alfalfa meal, and corn gluten meal [9,10]. In recent years, curcumin and lutein gradually replaced yellow corn and corn gluten meal becoming the most common natural dietary carotenoids in commercial poultry feed [8]. Lutein, 3 ′3 dihydroxy-α-carotene, is extracted from an abundant diversity of species of marigold [11]. And many monohydroxyl pigments occur in the metabolism and transformation of lutein including dehydrated lutein, β-cryptoxanthin, α-cryptoxanthin, and so on [12]. A large amount of research has resulted in lutein substituted synthetic pigment becoming a universal additive, which can enhance egg yolk color more effectively [13,14], and increase the yellow scores of skin and muscle of broilers [4,8]. However, there are few reports on the effects of monohydroxyl pigments, such as dehydrated lutein, β-cryptoxanthin, and α-cryptoxanthin on pigmentation in broilers.

Hence, this study was conducted to explore the differences in pigmentation between lutein and monohydroxyl pigments (dehydrated lutein, β-cryptoxanthin, and α-cryptoxanthin) extracted from marigold, in addition, we mixed lutein and monohydroxyl pigments in proportion in 1:1 to discover the function.

## MATERIALS AND METHODS

### Experimental animals and design

All experiments and procedures in this study were conducted according to the Animal Scientific Procedures Act 1986 (Home Office Code of Practice. HMSO: London January 1997) and EU regulations (Directive 2010/63/EU) and have been approved by the Institutional Animal Care and Use Committee of Wuhan Polytechnic University (No. WPU2022 11059). A total of 192 healthy 60-day-old broilers with an average body weight (BW) of 1,279±81 g were randomly divided into 4 groups with 6 replicates per group and 8 broilers per replicate. The 4 treatments were as follows: i) CON group, fed with basal diet; ii) LTN group, supplemented with lutein extracted from marigold; iii) MDP group, supplemented with monohydroxyl pigment extracted from marigold including dehydrated lutein, β-cryptoxanthin, and α-cryptoxanthin; iv) LTN+MDP group, supplemented with lutein and monohydroxyl pigment in proportion to 1:1. All treatments were supplemented with different types xanthophyll according to the ratio of 0.2%. The yellow scores of skin color was evaluated on day 7, 14, 21, and 28, and breast muscle, thigh muscle, and abdominal fat of slaughtered chickens were stored in cold storage at 4°C for 24 hours and taken out to determine the meat color of lightness (L*), redness (a*), and yellowness (b*) values. All chicks had free access to feed and water and were exposed to natural light and ventilation. The ambient temperature was maintained at 22 to 23 degrees Celsius with relative humidity controlled at 55% to 65%. The experiment lasted for 32 days, including 4 days of adaptation and 28 days of the experiment. The corn soybean meal-based basal diet formulated to meet the nutrition requirement for broilers is shown in Table 1, formulated according to the Poultry Nutrient Requirements [15]. The LTN, MDP, and the mixture of LTN+MDP were commercial products provided by Chen Guang Bio Co., Ltd, Hebei, China. The extraction of lutein from marigold is usually done with organic solvent extraction. According to the principle of similar phase dissolution, the solubility of lutein in different solvents is different, and the solvent with relatively high solubility is selected to dissolve lutein. Acid-catalyzed dehydration of lutein in a homogenous phase was carried out in a variety of solvents such as ethers, chlorinated solvents, acetone, and toluene at ambient temperature. Under reaction conditions, the monohydroxyl pigment including dehydrated lutein, β-cryptoxanthin, and α-cryptoxanthin was produced. The efficient content of LTN, MDP, and LTN+MDP is 1,662.8 ppm, 1,259 ppm, 841.4+629.2 ppm, respectively.

### Growth measurements

The health and death conditions of broilers were recorded every day during the trial period, and the BW and feed intake of broilers were determined on day 1 and day 28, respectively, and average daily gain (ADG), average daily feed intake (ADFI), and feed to gain ratio (F/G) were calculated.

### Skin color

Four chickens per replicate were randomly selected on day 7, 14, 21, and 28 during the trial period, and the yellow scores of shanks and beaks were evaluated by using the Kemin color fan obeying color scores from 1 to 14, according to previous research [2]. Skin color of the lateral subwing area of the chickens, including lightness (L*), redness (a*), and yellowness (b*) values, were measured using a Chromameter (CR-410, Konica Minolta, Tokyo, Japan).

### Carcass pigmentation

After sampling on the 28th day of the trial period, three chickens were randomly selected from each replicate for slaughtering, and breast muscle, thigh muscle, and abdominal fat were collected. Samples of slaughtered chickens were stored in cold storage at 4°C for 24 hours and taken out to determine the meat color of lightness (L*), redness (a*), and yellowness (b*) values of breast, thigh, and abdominal fat with a Chromameter calibrated with a whiteboard before measured. Each sample was measured three times at different sites and then its average value was taken.

### Statistical analysis

All data were analyzed by one-analysis of variance of SAS 9.1 software (SAS Inst. Inc., Cary, NC, USA) and presented as means and standard error of the mean. The pen was considered as the experimental unit for growth performance, and selected chickens that were slaughtered for sample collection were regarded as the experimental unit for skin color and carcass pigmentation. The significant level of data was declared at p<0.05. If significant effects were found, individual means were compared using Duncan’s multiple comparison tests.

## RESULTS

### Growth performance

As shown in Table 2, the BW on day 28 of the broilers supplemented MDP and LTN+MDP diets were not significantly different from the CON group (p>0.05), however, dietary supplementation LTN significantly decreased the BW compared to the CON group (p<0.05). Moreover, a similar consequence was shown in ADG. There was no significant difference in ADFI and F/G of treatment groups compared with the CON group (p>0.05).

### Skin color

**Figure 1:**
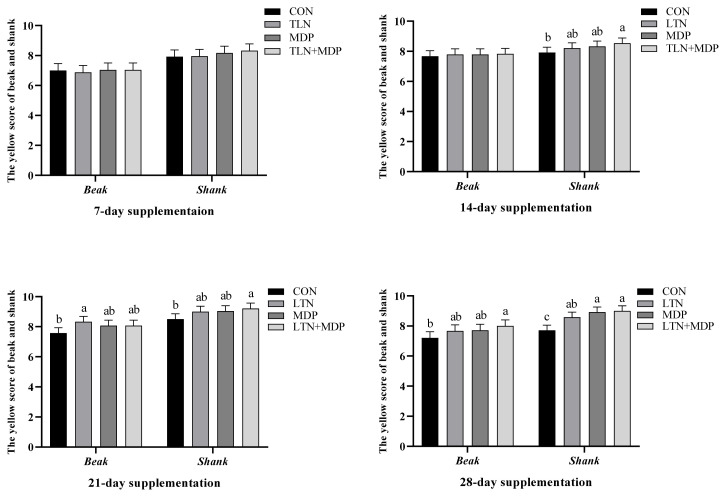
Effects of different types of xanthophyll extracted from marigold on the yellow scores of beaks and shanks in yellow-feathered chickens. CON, fed with basal diet; LTN, supplemented with lutein extracted from marigold; MDP, supplemented with monohydroxyl pigment extracted from marigold including dehydrated lutein, β-cryptoxanthin, and α-cryptoxanthin; LTN+MDP, supplemented with lutein and monohydroxyl pigment in proportion to 1:1. The statistics of the Y axis were measured, and the X axis presented the position of survey based on Kemin colour fan. Scores are mean and pooled standard error of the mean, n = 6 (1 bird per cage). ^a-c^ Different letters indicate significant differences between mean values for a given parameter (p<0.05).

**Figure 2:**
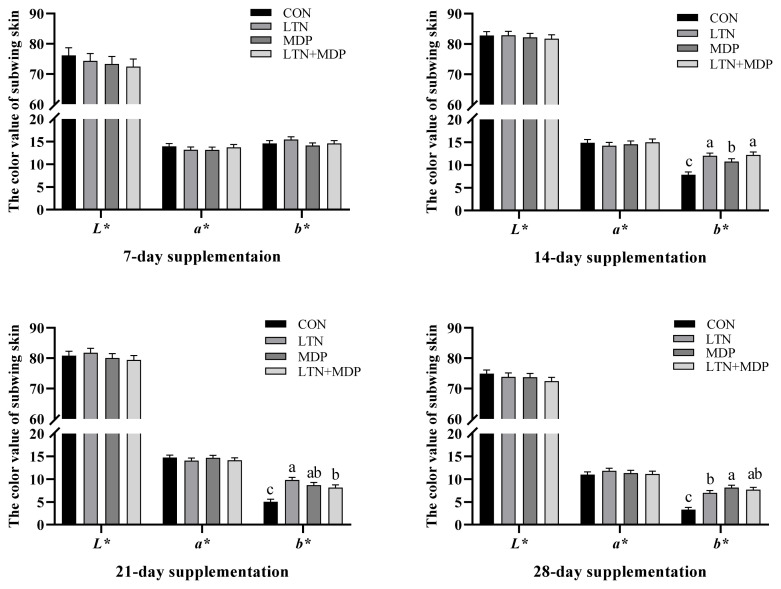
Effects of different types of xanthophyll extracted from marigold on the lightness (L*), redness (a*), and yellowness (b*) values of subwing skin in yellow-feathered chickens. CON, fed with basal diet; LTN, supplemented with lutein extracted from marigold; MDP, supplemented with monohydroxyl pigment extracted from marigold including dehydrated lutein, β-cryptoxanthin, and α-cryptoxanthin; LTN+MDP, supplemented with lutein and monohydroxyl pigment in proportion to 1:1. Scores are mean and pooled pooled standard error of the mean, n = 6 (1 bird per cage). ^a-c^ Different letters indicate significant differences between mean values for a given parameter (p<0.05).

No significant difference in the yellow scores of beaks and shanks was observed between all treatments and the CON group in the first week (p>0.05, Fugure 1). The analogous results in L*, a*, and b* values of subwing skin are presented in Figure 2 (p>0.05). After two weeks, the notable difference is shown in Figure 1 that the yellow scores of shank supplementing MDP and LTN+MDP diets were significantly enhanced over the CON group (p<0.05). In addition, the b* values of subwing skin in all treatments were significantly increased (p<0.05), and a better effect was exhibited in LTN and LTN +MDP groups than the MDP group in Figure 2 (p<0.05). As shown in Figure 1, in the third week, the LTN group significantly promoted the yellow scores of beaks, and the LTN+MDP group significantly enhanced the yellow scores of shanks (p<0.05). Moreover, the b* values of subwing skin in all treatments were significantly increased over the CON group in Figure 2 (p<0.05). Figure 1 and Figure 2 also demonstrates that after four weeks, supplementing with LTN+ MDP significantly increased the yellow scores of beaks, and the yellow scores of shank of all treatment groups (p<0.05). Furthermore, compared with the CON group, all treatment groups dramatically increased the b* values of subwing skin (p<0.05).

### Carcass pigmentation

As shown in Table 3, all treatment groups of LTN, MDP, and LTN-MDP significantly promoted the b* values of the breast muscle in comparison to the CON group (p<0.05). The skin results shown in Table 3 indicate that all treatments significantly enhanced the b* values of the thigh muscle (p<0.05). However, there was no significant difference in the L* and a* values of breast and thigh muscle between all treatments and the CON group (p>0.05). Additionally, Table 3 displays that the MDP and LTN+MDP groups dramatically increased the a* and b* values of abdominal fat more than the CON group (p<0.05).

## DISCUSSION

Carotenoids have become important pigment additives for improving animal products in poultry production [16,17], from the former yellow corn, dehydrated alfalfa meal, and corn gluten meal to the current common use of plant-derived curcumin, lutein and so on [18,19]. In corn-soybean meal-based diets, yellow corn contains about 17 ppm of xanthophyll, which is so low that additional colorants are needed to meet consumer preferences [20]. Particularly, lutein extracted from marigold is extensively used and there were many monohydroxyl pigments-dehydrated lutein, β-cryptoxanthin, and α-cryptoxanthin produced in the metabolic process [12]. A large amount of research has shown that carotenoids have no significant influence on growth performance. The addition of xanthophyll-rich okra meal had no significant difference in daily gain and the feed to gain ratio [2]. And the performance of pullets was not influenced by supplementing 4% marigold [21]. Our results indicated that compared with the CON group, the MDP and LTN+MDP had no significant difference in the BW of day 28 and the ADG, whereas the LTN group dramatically decreased the BW of day 28 and the ADG. Many studies have shown that lutein supplementation has no significant difference in growth performance. However, a few studies have suggested that large amounts of lutein may have negative effects on the liver. Supplementation with 400 mg/kg lutein has been shown to result in a decrease in serum erythrocyte count and hemoglobin content, as well as a decrease the relative organ weight of liver, and the presence of cystic tubular dilatation of kidneys, fatty change and accessory cortical nodules of the adrenal have been observed [22]. Moreover, studies have shown that aspartate aminotransferase and alanine aminotransferase significantly increased in a dose-dependent manner when lutein was added at 100 to 1,000 mg/kg [23]. Based on this consideration, it is speculated that a high dosage of lutein may affect liver health and lead to decrease the BW of day 28 and ADG.

The color of animal products, such as eggs, salmon and chicken, showed a close relationship with the preference of customers [24–26]. Genetic background, body state, and dietary pigment source and concentration affected the color of poultry to some extent, particularly, dietary pigment source and concentration [24]. It was reported that the gene associated with canthaxanthin and lutein was regarded as a decisive regulatory mechanism of pigmentation in broilers [27]. In this study, compared with the CON group, different lutein enhanced the b* values of subwing skin during the whole period, the LTN+MDP group increased the yellow scores of shank in the second week, the LTN treatment improved the yellow scores of beaks and the LTN+MDP treatment enhanced the yellow scores of shanks in the third week, and the LTN+ MDP treatment promoted the yellow scores of beaks and all groups boosted the yellow of shanks in the fourth week. Similar to our outcomes, in comparison to a basal diet, a diet supplemented with carotenoid notably boosted the yellow of the skin of broilers [28]. Consistent with our findings, it was demonstrated that supplementing lutein exhibited positive pigmentation efficiency of the skin yellow scores in the broiler [29]. Interestingly, the LTN+MDP group, mixing the lutein and monohydroxyl pigment including dehydrated lutein, β-cryptoxanthin, and α-cryptoxanthin displayed a more positive effect on skin pigmentation. The possible reason for this result is that dehydratied lutein, β-cryptoxanthin, and α-cryptoxanthin, which are the metabolizing and transiting products of lutein, are better absorbed and deposited in tissues than lutein.

Various factors determine meat quality such as meat color, drip loss, cooking loss, and shear force, yet color is crucial to the appearance of meat and consumer preference [30,31]. Carotenoids have been used to improve the meat color of poultry, due to the characteristic of amassing in the body [32]. The results of this research showed that dietary supplements different types of xanthophyll enhanced the b* values of breast and thigh muscle, and the MDP and LTN+MDP groups increased the a* and b* values of abdominal fat in comparison to the CON group. Consistent with our results, the addition of xanthophyll-rich okea meal to the diet increased the b* values of breast and abdomen fat [2]. Many works have indicated xanthophyll extracted from marigold was more efficient deposition in the muscle and skin than synthetic pigment [28,29,33]. Moreover, the dietary supplement of marigold extract enhanced the yellowness values of carcass and meat quality [34]. However, the mechanism is not clear that increasing the a* value of abdominal fat caused by supplementing with monohydroxyl pigment or lutein and monohydroxyl pigment in proportion to 1:1.

## CONCLUSION

In conclusion, different types of xanthophyll extracted from marigold including lutein, monohydroxyl pigment (dehydrated lutein, β-cryptoxanthin, and α-cryptoxanthin), and the mixture of lutein and monohydroxyl pigment can enhance the yellow scores of beaks, shanks and subwing skin, and increase the b* values of beast, thigh and abdomen fat to improve meat quality. Especially, the mixture of lutein and monohydroxyl pigment is more efficient on skin color.

## Figures and Tables

**Table 1:** Composition and nutrition levels of the basal diet

Item	Ratio
Ingredients (%)	
Corn	60.58
Soybean meal, 44% crude protein	32.00
Soybean oil	3.00
Calcium hydrophosphate	1.20
Limestone	1.20
Salt	0.30
L-Lysine	0.35
DL-Methionine	0.22
L-Threonine	0.15
Premix^1)^	1.00
Total	100.00
Nutrient levels^2)^	
ME (kcal/kg)	3,100
Crude protein (%)	16.4
Calcium (%)	0.81
Available phosphorus (%)	0.35
Lysine (%)	0.72
Methionine + cystine (%)	0.49
Threonine (%)	0.54
Tryptophan (%)	0.17

ME, metabolizable energy.

1) Premix supplied per kg diet: vitamin A, 12,500 IU; vitamin D_3_, 2,600 IU; vitamin E, 24 mg; vitamin K_3_, 4 mg; riboflavin, 5 mg; D-calcium pantothenate, 24 mg; nicotinic acid, 55 mg; cobalamin, 0.03 mg; manganese (MnSO_4_·H_2_O), 55 mg; iron (FeSO_4_·H_2_O), 75 mg; zinc (ZnSO_4_·7H_2_O), 36 mg; copper (CuSO_4_·5H_2_O), 8 mg; selenium (Na_2_SeO_3_), 0.15 mg.

2) The nutrients contents of metabolizable energy and available phosphorus were calculated values, and lysine, methionine + cystine, threonine, crude protein and calcium content were analytical value.

**Table 2:** Effects of different types of xanthophyll extracted from marigold on the growth performance

Item	Treatments^1)^				SEM	p-value

	CON	LTN	MDP	LTN+MDP		
D 1 BW (g)	1,261	1,265	1,275	1,281	21	0.856
D 28 BW (g)	1,897^ab^	1,809^c^	1,884^ab^	1,922^a^	25	0.025
ADG (g/d)	22.7^a^	19.4^b^	21.8^a^	22.9^a^	1.1	0.008
ADFI (g/d)	103.4	98.7	104.9	108.8	5.75	0.126
F/G	4.56	5.09	4.81	4.75	0.42	0.652

n = 8 (1 bird per cage).

SEM, standard error of the mean; BW, body weight; ADG, average daily gain; ADFI, average daily feed intake; F/G, feed to gain ratio.

1) CON, fed with basal diet; LTN, supplemented with lutein; MDP, supplemented with monohydroxyl pigment including dehydrated lutein, β-cryptoxanthin, and α-cryptoxanthin; LTN+MDP, supplemented with lutein and monohydroxyl pigment in proportion to 1:1.

a–c Means with different small letters within a row differ (p<0.05).

**Table 3:** Effect of different types of xanthophyll extracted from marigold on the lightness (L*), redness (a*), and yellowness (b*) values of breast, thigh muscle, and abdominal fat in yellow-feathered chickens

Item	Treatments^1)^				SEM	p-value

	CON	LTN	MDP	LTN+MDP		
Breast						
L*	74.02	74.70	76.11	75.17	2.22	0.269
a*	10.96	9.91	10.74	10.44	1.03	0.335
b*	9.28^b^	12.48^a^	11.74^a^	13.74^a^	1.21	<0.001
Thigh						
L*	83.06	79.52	80.81	79.39	2.46	0.762
a*	14.65	15.26	15.20	15.65	0.88	0.726
b*	9.26^b^	13.96^a^	13.76^a^	14.30^a^	1.12	<0.001
Abdominal fat						
L*	96.11	96.57	97.22	96.67	0.85	0.885
a*	6.48^b^	7.72^ab^	8.80^a^	8.69^a^	0.62	<0.001
b*	26.91^b^	31.04^ab^	32.41^a^	35.46^a^	2.31	<0.001

Scores are mean and pooled standard error of the mean, n = 6 (1 bird per cage).

1) CON, fed with basal diet; LTN, supplemented with lutein extracted from marigold; MDP, supplemented with monohydroxyl pigment extracted from marigold including dehydrated lutein, β-cryptoxanthin, and α-cryptoxanthin; LTN+MDP, supplemented with lutein and monohydroxyl pigment in proportion to 1:1.

a,b Different letters indicate significant differences between mean values for a given parameter (p<0.05).
